# Macrolides and associated antibiotics based on similar mechanism of action like lincosamides in malaria

**DOI:** 10.1186/s12936-016-1114-z

**Published:** 2016-02-12

**Authors:** Tiphaine Gaillard, Jérôme Dormoi, Marylin Madamet, Bruno Pradines

**Affiliations:** Unité de Parasitologie, Département d’Infectiologie de Terrain, Institut de Recherche Biomédicale des Armées, Marseille, France; Unité de Recherche sur les Maladies Infectieuses et Tropicales Emergentes, Aix Marseille Université, UM 63, CNRS 7278, IRD 198, Inserm, 1095 Marseille, France; Fédération des Laboratoires, Hôpital d’Instruction des Armées Saint Anne, Toulon, France; Unité de Parasitologie et d’Entomologie, Département des Maladies Infectieuses, Institut de Recherche Biomédicale des Armées, Brétigny sur Orge, France; Equipe Résidente de Recherche en Infectiologie Tropicale, Institut de Recherche Biomédicale des Armées, Hôpital d’Instruction des Armées, Marseille, France; Centre National de Référence du Paludisme, Marseille, France

**Keywords:** Antibiotics, Antimalarial drug, Malaria, *Plasmodium falciparum*, Macrolides, Lincosamides, Treatment, Resistance

## Abstract

Malaria, a parasite vector-borne disease, is one of the biggest health threats in tropical regions, despite the availability of malaria chemoprophylaxis. The emergence and rapid extension of *Plasmodium falciparum* resistance to various anti-malarial drugs has gradually limited the potential malaria therapeutics available to clinicians. In this context, macrolides and associated antibiotics based on similar mechanism of action like lincosamides constitute an interesting alternative in the treatment of malaria. These molecules, whose action spectrum is similar to that of tetracyclines, are typically administered to children and pregnant women. Recent studies have examined the effects of azithromycin and the lincosamide clindamycin, on isolates from different continents. Azithromycin and clindamycin are effective and well tolerated in the treatment of uncomplicated malaria in combination with quinine. This literature review assesses the roles of macrolides and lincosamides in the prophylaxis and treatment of malaria.

## Background

Malaria, a parasite vector-borne disease, is one of the largest health threats in tropical regions, despite the availability of malaria chemoprophylaxis and the use of repellents and insecticide-treated nets [1]. The prophylaxis and chemotherapy of malaria remains a major area of malaria research, and new molecules are constantly being developed prior to the emergence of resistant parasite strains. The use of anti-malarial drugs is conditioned based on the level of resistance of *Plasmodium falciparum* in endemic areas and the contraindications, clinical tolerance and financial costs of these drugs. Among the compounds potentially used against *Plasmodium*, antibiotics have been examined in vitro or in vivo.

After tetracyclines, the second family of potential antibiotics in the fight against *Plasmodium* includes macrolides and macrolide derivatives, a class of compounds with 14-20 membered macrolactone ring. Another class of antibiotics, licosamides whose chemical structure differs from the macrolides, are associated with the macrolides based on similar mechanism of action. Recent studies have examined the effects of the macrolide azithromycin and the lincosamide clindamycin, on isolates from different continents [2–4]. These molecules, whose action spectrum is similar to that of tetracyclines, are typically administered to children and pregnant women [5–11].

This literature review assesses the roles of macrolides and macrolide derivatives in the prophylaxis and treatment of malaria.

### Classification of macrolides and associated antibiotics based on similar mechanism of action like lincosamides

The macrolide family comprises lincosamides and streptogramines, referred to as the MLSB group. These antibiotics, with a distinct chemical structure, are classified in the same group based on comparable activity spectra and identical functional mechanism, based on the inhibition of protein synthesis. These antibiotics, with limited activity spectra, are particularly active against the intracellular germs and are currently administered to children and pregnant women [5–11], conferring a notable advantage compared with tetracyclines. Certain antibiotics of this family are useful as anti-malarial substances.

The classification of macrolides is based on the number of carbon links, which distinguishes macrolides with 14 atoms from 15- or 16-membered macrolides. The 14-membered erythromycin is the oldest molecule (1952); all second-generation macrolides: roxithromycin, clarithromycin and dirithromycin [12] are products of hemisynthesis and derived from erythromycin. The only azalide with 15 carbon atoms is azithromycin, produced through the introduction of a nitrogen atom inserted into the macrolide nucleus at C10. This modification has improved the penetration of drugs into macrophages, fibroblasts and polymorphonuclear neutrophils, facilitating increased accumulation within acidified vacuoles and extending the half-life [4]. It has also improved activity against Gram-negative bacteria and other pathogens from parasitic infections [13]. The antibiotics with 16 carbon links are spiramycin and josamycin. Chemical modifications are constantly being developed to optimize this family [14].

Lincosamides are antibiotics associated with the macrolides based on a similar action spectrum, although the structure of these compounds differs. Lincomycin is a sugar isolated in 1962 from fermentation through *Streptomyces lincolnensis*. Substitution of the C7 bearing a hydroxyl function with a chlorine atom generated a semi-synthetic derivative, such as 7-chloro-7-deoxy-lincomycin or clindamycin, with a higher antibiotic activity and digestive absorption. Clinical trials have shown the efficacity, safety and practicability of the treatment of *P. falciparum* malaria with clindamycin [2].

### Pharmacological properties

Macrolides exhibit poor digestive absorption of less than 60 %, and this effect is strongly influenced by food. The half-life of these drugs is variable, from a short half-life for erythromycin (2 h) and clarithromycin (4 h) to long half-life for azithromycin (68 h) [3]. Efforts for development of molecules of this family were first employed to improve pharmacological properties, but not antimicrobial activities.

With the exception of cerebrospinal fluid and brain, macrolides show excellent tissue distribution in tissues, such as bone and liver. Erythromycin and clarithromycin are highly metabolized in the liver through interactions with cytochrome P450 CYP3A4, which has been implicated in many drug interactions. The elimination of macrolide and macrolide derivatives is primarily biliary. Indeed, liver failure severely disrupts pharmacokinetics. Concerning azithromycin, mild renal dysfunction and mild-to-moderate hepatic dysfunction do not significantly affect excretion.

Azithromycin is a slightly metabolized molecule, with 37 % bioavailability after oral administration [15]. It is known to have a large volume of distribution, achieving high tissue concentrations. It accumulates in hepatic, renal, pulmonary and splenic tissues and gradually leaches into the bloodstream over a period of 1 week [16]. It also accumulates within fibroblastes and phagocytic lysosomes; these cells may serve as a reservoir for slow release of the drug [17]. Compared with older generation macrolides, it is more stable in acidic media and has a longer half-life, allowing for once a day [18]. Indeed, following a standard 500 mg oral dose, peak plasma concentrations are attained with a Tmax of 2–4 h; the plasma half-life of azithromycin is approximately 70 h following oral formulation [18].

Among lincosamides, clindamycin has a 90 % digestive absorption. Unlike lincomycin, clindamycin absorption is not reduced, but only delayed, through food intake. Characterized by slow but thorough anti-malarial activity, clindamycin presents a remarkably short plasma half-life (2–4 h) [7]. Lincosamides exhibit good tissue penetration, and contrary to macrolides, pass the blood–brain barrier. Clindamycin metabolism occurs in the liver, with elimination and high bile concentration. In hepatic insufficiency, the half-life can be extended twice and doses should be reduced accordingly. With respect to the effect on *Plasmodium*, clindamycin slowly accumulates in parasites [6].

### Tolerance

Macrolides are generally well tolerated; moreover, they exhibit a good safety profile in children and pregnant women [14]. The adverse events most frequently reported are gastrointestinal disorders: nausea, vomiting, and episgastralgia associated with the administered dose. Other side effects, such as neurosensory in the type of headache and dizziness disorders, skin allergies and rare cases of cholestatic hepatitis, have been reported, but these effects are rare. The “old” molecules typically present more side effects than the newer molecules. Macrolides are largely a problem of drug interference, reflecting the role of cytochrome [19]. Notably, although the risk of drug interactions is high, the risk of interactions with new molecules is much less important.

Drug interactions are less frequently observed with lincosamides than with macrolides. The oral forms of lincosamides exhibit a more irritative effect (esophagitis) than the parenteral forms (chemically induced phlebitis). Systemic reactions, including allergies, skin reactions and anaphylactic shock, have been reported. Diarrhoea and digestive disorders primarily occur with the oral forms [2]. Moreover, the appearance of pseudomembranous colitis resulting from *Clostridium difficile* toxin selection is characterized by profuse watery diarrhoea, fever, and occasional bleeding, requiring the discontinuation of treatment [20]. Moreover, rapid intravenous administration might reflect the electrocardiographic changes and even collapse of cardiac arrest observed in response to lincosamide treatments [21]. Haematologic disorders, such as leukopenia, neutropenia, and thrombocytopenia have been reported. Gastrointestinal disorders are frequently reported in patients receiving azithromycin. Doses of azithromycin between 500 and 2000 mg have been used in all trimesters of human pregnancy for the treatment of upper and lower respiratory tract infections, skin diseases, and infections with *Chlamydia trachomatis*, *Mycoplasma* and group B streptococci among women allergic to other antibiotics [4]. Nevertheless, azithromycin delays cardiac polarization [21, 22], although preliminary studies concerning the combination of azithromycin with chloroquine for QT prolongation indicate that cardiac instability is not increased under this combination [23].

### Mechanism of antiplasmodial action

In bacteria, macrolides inhibit the synthesis of cell proteins through binding to the 50S subunit of the ribosome. The inhibition of protein synthesis through the inhibition of transpeptidation explains the postantibiotic effects of this drug, measured after 3–4 h. The macrolide antibacterial spectrum is similar to that of erythromycin. This spectrum is limited to Gram-positive bacteria, and Gram-negative bacilli remain impermeable to these molecules; however, because of the intracellular concentration of these drugs, macrolides are active against intracellular bacteria development [24]. New macrolide compounds, including the azalide azithromycin, and lincosamides present more or less broader antibacterial activity than erythromycin; the lincosamide clindamycin remains of particular interest as therapy for some parasitic infections [13]. Anti-malarial properties occur by targeting the bacterium-derived translational machinery in the relict plastid, apicoplast, present in *Plasmodium* spp. [14]. This organelle is limited by four membranes and located within parasitic cells; it contains a 35 kb circular DNA allowing [16] replication, RNA transcription and RNA–protein translation [25].

The macrolide antibiotic azithromycin exhibits the best antiplasmodial properties. This molecule targets the 70S ribosomal subunit from the apical complex [16], comprising 50S and 30S subunits. Once fixed, macrolide prevents the synthesis of the polypeptide, which is subsequently prematurely released [4]. The synthesis of a nonfunctional apicoplast, resulting from exposure to azithromycin, is at the origin of the delayed effect of the molecule. Indeed, parasites treated during first 48 h life cycle show non obvious defect from the loss of apicoplast-encoded gene products: organelle morphology, genome replication protein targeting and segregation during cell division remain intact. Likewise, parasites progress normally through the different developmental stages, giving rise to daughter merozoites that successfully reinvade to establish infection of a new host cell. The deleterious effects of antibiotic occur in the second life cycle following antibiotic treatment in which the apicoplast genome fails to replicate [26]. Thus, similar to tetracyclines, the antiplasmodial action of this macrolide is therefore delayed [27, 28]; this phenomenon in which the parasite completes a full cycle before achieving growth inhibition is referred to “delayed death” [29]. Delayed death is a strategy for examining whether an antibiotic acts on the apicoplast, and unlike antiparasitic molecules with immediate effects, the activity of antiparasitic compounds on some functions of the apicoplast is measurable beyond cell division. Several studies have also identified the immediate activity of azithromycin [30–32], well above that of older macrolides. The mechanism responsible for this activity has not been elucidated [14] and clinical studies failed to demonstrate even equivalence of 3-day treatment with azithromycin to other anti-malarial drugs [18].

The target of clindamycin has been recently demonstrated in *Plasmodium*. This drug was originally extrapolated from *Toxoplasma gondii*, frequently used as a model based on structural similarities [2, 33]. In *T. gondii*, clindamycin and the three major clindamycin metabolites are fixed to the large subunit ribosomal RNA of the apicoplast [33]. Several studies have shown a lethal effect of clindamycin on potentiated parasites after 72 h of exposure [29], although the antibiotic concentrations were reduced 3–4 factors less than the IC_50_.

It has also been suggested that parasites exposed to clindamycin divide and invade new host cells, but at this point, the cells are unable to grow and eventually perish. These results, prior to an in-depth study of the apicoplast, revealed the toxicity of clindamycin on a structure involved in the translation of plasmodial ribosomal RNA into protein [34]. These findings contributed to the antiplasmodial action of clindamycin after 3 days of administration [35]. In 2005, the target of clindamycin was identified [36]. Clindamycin binds the 50S subunit, comprising ribosomal 23S, 5S and ribosomal proteins L4 and L22. The same mode of action was described for azithromycin two years later. *Plasmodium falciparum* ribosomal protein L4 (PfRpl4) has been demonstrated to associate with the nuclear genome-encoded *P. falciparum* ribosomal protein L22 (PfRpl22) and the large subunit rRNA 23S to form the 50S ribosome polypeptide exit tunnel, which could be occupied by azithromycin [16].

### Clinical effectiveness

Due to the short half-life of the first generation of macrolides, their use for anti-malarial treatment is limited. The best-studied antiplasmodial molecules include azithromycin, for which chemical modifications significantly increase the half-life, and clindamycin.

In this section, the clinical trials using azithromycin and those using clindamycin will be successively discussed.

Concerning azithromycin, its antiplasmodial action was first described in vitro at the beginning of the 90s [17, 37]. At the end of the 90s, the mass distribution of azithromycin through the World Health Organization (WHO) trachoma elimination programme was shown to reduce malarial parasitaemia [38]. Several studies concerning the antiparasitic properties of antibiotics showed the delayed action of the molecule [16, 27, 28]. Only one clinical multicentre study of azithromycin for the treatment of acute uncomplicated *P. falciparum* malaria was conducted in India on 15 participants. In this study, patients were randomly assigned to groups treated with either azithromycin or chloroquine alone, or azithromycin associated with chloroquine [3]. The resolution of parasitaemia was inadequate with monotherapy with either azithromycin or chloroquine, but combination therapy provided substantially improved clinical and parasitological outcomes. The delayed resolution of parasitaemia and the potential adverse effects that may occur with effective high doses [39] confirmed that this drug was unsuitable for monotherapy treatment by azithromycin. In addition, different associations were tested in vivo (Table 1).

**Table 1:** Clinical trials of azithromycin plus other drug against *P. falciparum* malaria

Study demographic details					Regimen								Efficacy ( %) d28
Year	Place	Reference	Pop	Nb	Azithromycin			Other Drug			Route	Days	
					Dosage/d	Route	Nb doses/d	Drug^a^	Dosage/d	Nb doses/d			
1996	Thailand	NaBangchang [78]	A	30	500 mg	PO	1	Ath	300 mg	1	PO	3/1	14.8
2001	India	Dunne 2005 [3]	A	64	1000 mg	PO	2	C	600 mg	2	PO	3	90
2006	Thailand	Noedl 2006 [41]	A	27	1500 mg	PO	2	A	200 mg	2	PO	3	92
			A	27	1000 mg	PO	1	A	200 mg	1	PO	3	89
			A	16	1500 mg	PO	2	Q	20 mg/kg	2	PO	3	73
			A	27	1500 mg	PO	3	Q	30 mg/kg	3	PO	3	92
2006	Thailand	Miller 2006 [44]	A	10	1000 mg	PO	2	Q	30 mg/kg	3	PO	3	90
			A	20	1000 mg	PO	2	Q	30 mg/kg	3	PO	5	100
			A	20	1500 mg	PO	3	Q	30 mg/kg	3	PO	3	100
2007	Malawi	Kalilani 2007 [8]	P	47	1000 mg	PO	2	SP	1500/75 mg	3	PO	2	91
2008	Tanzania	Sykes 2009 [9]	C	129	20 mg/kg	PO	1	A	4 mg/kg	1	PO	3	42
2009	Bangladesh	Thriemer 2010 [43]	A		1500 mg	PO	1	A	200 mg	1	PO	3	95
			or	152	or				or				
			C		30 mg/kg	PO	1	A	4 mg/kg	1	PO	3	95
2007–8	Malawi	Laufer 2012 [10]	C	160	30 mg/kg	PO	1	C	10 mg/kg	1	PO	2	99
2004–6	Africa	Sagara 2014 [11]	A	227	1000 mg	PO	1	C	600 mg	1	PO	3	99

Randomized controlled trial

Only trials with adequate dosing, i.e. clindamycin given at least twice daily are mentioned in this table

*Pop* population, *A* adult, *C* children, *P* pregnant women

^a^ *A* artesunate, *Ath* arthemeter, *C* chloroquine, *F* fosmidomycin, *Q* quinine, *SP* sulfadoxine–pyriméthamine

The effects of associations, such as azithromycin–chloroquine and azithromycin–quinine, were additive on sensitive chloroquine strains and synergistic on resistant strains [40]. Other associations were examined, showing effectiveness, associating azithromycin with a rapidly acting schizonticidal compounds, such as lumefantrine or artemisinin [9, 41]. Two in vitro studies [40, 42] suggested that the dihydroartemisinin-azithromycin combination had antagonistic effects and should be avoided. An in vivo study conducted in Thailand [41], a geographic area with high levels of resistance to anti-malarial drugs, showed that azithromycin–artesunate, even when administered only once daily for 3 days, and azithromycin–quinine, administered three times daily, are safe and efficacious combination treatments for uncomplicated falciparum malaria. A randomized controlled trial performed in Tanzanian children did not support the use of azithromycin–artesunate as treatment for malaria; indeed, the 58 % parasitological failure rate observed after day 28 clearly showed that this treatment could not be an appropriate first line treatment for malaria [9]. One clinical trial conducted in Bangladesh performed on 152 patients suggested that this combination was an efficacious and well-tolerated treatment for patients with uncomplicated falciparum malaria compared with the artemether–lumefantrine combination [43]. This study did not consider the re-emergence of parasites in the peripheral blood as a failure of the treatment, although the mean time was 31.5 ± 5 days. Moreover, these authors did not distinguish the study group according to the age of the patients and mixed children and adults for the data integration.

The efficacy of the azithromycin–quinine combination was confirmed in 2006 [44] when 100 % of the patients were cured through high azithromycin regimens (combination of quinine with 1000 mg of azithromycin per day for 5 days or 1500 mg of azithromycin for 3 days).

A longitudinal trial comparing the effects of chloroquine as a monotherapy or in combination with other drugs, including azithromycin, on children with repeated malaria infections in Malawi demonstrated a high efficacy of the repeated administration of different regimens and showed a significantly higher haemoglobin concentration in children in the chloroquine–azithromycin group. This result might reflect the prevention or treatment of bacterial infections [10]. This combination, chloroquine–azithromycin was recently confirmed as highly efficient and well tolerated in African adults [11].

Another combination treatment comprising azithromycin with sulfadoxine–pyrimethamine was tested in pregnant women from Malawi [8]. Sulfadoxine–pyrimethamine has been adopted in many sub-Saharan Africa countries as the drug of choice for intermittent preventive therapy to reduce placental malaria and low-birth weight. The azithromycin–sulfadoxine–pyrimethamine combination might have several advantages: first, although the parasite clearance rate was slow compared with sulfadoxine–pyrimethamine–artesunate, the rate of recrudescence was low and markedly similar between the two groups. Secondly, azithromycin has an adequate safety profile, as this molecule has often been used in pregnant women to treat STIs. In contrast, there has been concern about the use of artemisinin derivatives during the first trimester based on animal studies [45]. Thirdly, azithromycin has a relatively long half-life compared with artesunate. The azithromycin–sulfadoxine–pyrimethamine combination protects the longer-acting drug (sulfadoxine–pyrimethamine) [8], by decreasing the probability of parasites encountering sub-therapeutic drug levels and promoting the development of resistance [46].

Despite these results, a review from the Cochrane Collaboration [39] concluded that the available evidence suggested that azithromycin was a weak anti-malarial with some appealing safety characteristics, and that azithromycin’s future for the treatment of malaria did not look promising.

Concerning lincosamides, clindamycin is a major antibiotic for the treatment of anaerobic bacterial infections [47]. This drug also presents antimicrobial activity against *Plasmodium*, *Toxoplasma*, *Babesia* and *Pneumocystis spp*. Moreover, clindamycin is the drug of choice for treatment against toxoplasmic chorioretinitis in newborns and one of the treatments recommended in the babesiosis with *Babesia microti* and *B. divergens* [48]. Associated with pyrimethamine or primaquine, clindamycin is a treatment of second intention against toxoplasmosis and pneumocystosis [49].

The antiplasmodial indication of clindamycin was managed according to various therapeutic regimens. The effectiveness of clindamycin in monotherapy in this indication was initially reported in 1975 [50]. The WHO repeated this protocol in several studies conducted on different continents, and several sightings have been reported (Table 2), including the effectiveness of clindamycin in monotherapy against malaria. This efficiency is however conditioned through treatment for 5 days, with twice-daily administration, as this molecule acts slowly. Clindamycin is well tolerated, and minor side effects have been reported during treatment. The occurrence of diarrhoea resulting from *Clostridium difficile* has often been reported after treatment with clindamycin, and this side effect might progress to pseudomembranous colitis, as a result of lengthy treatment with antibiotics [51]. The potential problem of severe diarrhoea, observed in patients receiving a prolonged and high dose of clindamycin therapy, is not observed with a low dose and short duration of therapy to treat malaria [52]. The WHO did not ultimately recommend clindamycin treatment when used alone as an anti-malarial treatment, as parasite clearance might be deleterious in cases of significant parasitaemia in fragile subjects (children and pregnant woman) [2]. However, clindamycin is now recommended for pregnant women in the first trimester with uncomplicated malaria, in association with quinine or artemisinin-based combination therapies or oral artesunate for 7 days.

**Table 2:** Clinical trials of clindamycin monotherapy against *P. falciparum* malaria

Study demographic details					Regimen					
Year	Place	Reference	Pop	Nb	Dosage	Form	Nb doses/d	Route	Nb days	Efficacy (%)
1975	USA	Clyde, 1975 [50]	A	3	450 mg	Salt	3	PO	3	100
1975	Thailand	Hall, 1975 [79]	A	10	450 mg	Salt	3	PO	3	50
1981	Brazil	Alecrim, 1981 [80]	A	17	10 mg/kg	Salt	2	IV	3	65
1981	Brazil	Alecrim, 1981 [80]	A	14	10 mg/kg	Salt	2	IV + PO	7	100
1982	Brazil	Alecrim, 1982 [81]	A	26	10 mg/kg	Salt	2	IV, PO	5	100
1982	Philippines	Rivera, 1982 [82]	A	24	300 mg	Salt	2	IV + PO	7	100
1982	Philippines	Rivera, 1982 [82]	A	12	600 mg	Salt	2	IV + PO	7	100
1982	Philippines	Cabrera, 1982 [83]	A	12	10 mg/kg	Salt	2	IV + PO	7	100
1982	Philippines	Cabrera, 1982 [83]	A	19	20 mg/kg	Salt	2	IV	3	89
1984	Columbia	Restrepo, 1984 [84]	A	6	20 mg/kg	Salt	2	IV	3	100
1984	Columbia	Restrepo, 1984 [84]	A	9	10 mg/kg	Salt	2	IV + PO	7	100
1984	Columbia	Restrepo, 1984 [84]	A	5	20 mg/kg	Salt	2	IV	7	100
1984	Columbia	Restrepo, 1984 [84]	A	10	20 mg/kg	Salt	1	IV	7	100
1985	Sudan	El Wakeel, 1985 [85]	A	20	5 mg/kg	Salt	2	PO	5	90
1988	Brazil	Meira, 1988 [86]	A, C	129	10 mg/kg	Salt	2	PO, IV	5–7	97
1988	Brazil	Meira, 1988 [86]	A, C	16	10 mg/kg	Salt	1	PO, IV	5–7	50
1988	Brazil	Meira, 1988 [86]	A, C	35	2.5 mg/kg	Salt	1	PO	5	80
1989	Brazil	Kremsner, 1988 [20]	A	35	5 mg/kg	Base	2	PO	5	100
1990	Philippines	Salazar, 1990 [87]	A	31	300 mg	Salt	2	PO	5	100
1990	Philippines	Salazar, 1990 [87]	A	10	600 mg	Salt	2	PO	5	100
1993	Gabon	Salazar, 1990 [87]	A	38	5 mg/kg	Base	2	PO	5	97
1994	East Timor	Oemijati, 1994 [88]	A	30	300 mg	Salt	2	PO	5	100

*A* adults, *C* children

The combination of clindamycin with other rapidly acting drugs is essential for the optimization of treatment. Clinically documented associations essentially involve the combination of clindamycin with quinine or chloroquine.

Quinine, showing a rapid onset and short half-life, is the ideal partner. In vitro studies have also shown a synergistic effect when the two molecules are associated [7, 52]. The bioavailability of the two drugs, when co-administered, remains unchanged [53]. A methodology and satisfactory post-treatment follow-up in approximately ten clinical trials with a wide number of patients have been published (Table 3) [2]. The duration of combination therapy remains controversial. While most studies consider that the administration of quinine for at least 7 days and clindamycin for at least 5 days is needed, treatments conducted for 3 days in African studies were effective [52, 54]. Short-duration treatment is justified for obtaining adequate compliance and fear of side effects with quinine. Parasite clearance has been correlated with parasitaemia in children treated for 4 days [55, 56]. In areas of multidrug resistance, such as Thailand, 5–7 days are needed to cure malaria.

**Table 3:** Clinical trials of clindamycin plus other drug against *P. falciparum* malaria

Study demographic details					Regimen								Efficacy (%)
Year	Place	Reference	Pop	Nb	Clindamycin			Other drug			Route	Days	
					Dosage	Form	Nb doses/d	Drug^a^	Dosage/d	Nb doses/d			
1974	USA	Miller, 1973 [53]	A	5							PO	3	100
1975	USA	Clyde, 1975 [50]	A	5	450 mg	Salt	3	Q	560 mg	3	PO	3	60
1975	USA	Clyde, 1975 [50]	A	2	600 mg	Salt	1	Q	560 mg	3	PO	3	50
1975	Thailand	Hall, 1975 [79]	A	4	450 mg	Salt	3	Q	540 mg	3	PO	3	100
1975	Thailand	Hall, 1975 [79]	A	5	150 mg	Salt	3	Q	270 mg	3	PO	3	60
1987	Brazil	Kremsner, 1988 [20]	A	40	15 mg/kg	Base	2	Q	10 mg/kg	2	PO	3	90
1992	Gabon	Kremsner, 1994 [52]	C	34	5 mg/kg	Base	2	Q	12 mg/kg	2	PO	3	88
1993	Gabon	Metzger, 1995 [89]	C	33	5 mg/kg	Base	2	CQ	25 mg/kg	3	PO	3	70
1995	Gabon	Kremsner, 1995 [90]	C	50	5 mg/kg	Base	3	Q	8 mg/kg	3	IV	4	96
1995	Gabon	Metzger, 1995 [54]	A	40	5 mg/kg	Base	2	Q	12 mg/kg	2	PO	3	92
1996	France	Parola, 2001 [91]	A	53	5 mg/kg	Salt	3	Q	8 mg/kg	3	IV	3	100
1997	Gabon	Vaillant, 1997 [57]	C	161	8 mg/kg	Salt	2	Q	8 mg/kg	2	PO	3	97
2000	Thailand	Pukrittayakamee [92]	A	68	5 mg/kg	Base	4	Q	8 mg/kg	3	PO	7	100
2001	Thailand	McGready, 2001 [5]	P	65	5 mg/kg	Salt	3	Q	8 mg/kg	3	PO	7	100
2004	Gabon	Bormann, 2004 [6]	C	12	10 mg/kg	Salt	2	F	60 mg/kg	2	PO	5	100
2004	Gabon	Ramharter, 2005 [7]	C	100	7 mg/kg	Salt	2	A	2 mg/kg	2	PO	3	87

Randomized controlled trial

Only trials with adequate dosing, i.e. clindamycin given at least twice daily are mentioned in this table

*Pop* population, *A* adult, *C* children, *P* pregnant women

^a^ *C* chloroquine, *Q* quinine, *F* fosmidomycin, *A* artesunate

The second well-studied combination is clindamycin with chloroquine. *Plasmodium falciparum* is highly resistant to chloroquine in most malarial regions. However, this drug is still widely used and remains a first-line treatment in Africa. The clindamycin–chloroquine combination has been studied in Gabon [52], where chloroquine resistance is markedly high. Clindamycin was administered every 12 h for 3 days, and success rates ranged from 70 % in children to 97 % in adults, depending on the study [57]. The success rate in children was estimated as 94 % with chloroquine administered at a dose of 45 versus 25 mg/kg. Although these findings favour the effectiveness of the combined administration of chloroquine with clindamycin for 3 days, this treatment has not been widely adopted in practice.

Fosmidomycin, a phosphonic acid derivative, is a new anti-malarial drug with a novel mechanism of action that inhibits the synthesis of isoprenoid in *P. falciparum* and suppresses the growth of multidrug-resistant strains in vitro [58]. Studies in Africa evaluating fosmidomycin as a monotherapeutic agent demonstrated that the drug is well tolerated in humans. A randomized, controlled, open-label study was conducted in 2003 in children to evaluate the efficacy and safety of treatment with fosmidomycin combined with clindamycin (30 and 5 mg/kg body weight every 12 h for 5 days, respectively) compared with treatment with either fosmidomycin or clindamycin alone. The combined treatment with the two molecules was superior to that with either agent alone [6].

Since 2010, the WHO advocates artemisinin-based combination therapy (ACT) as the mainstay in combating drug-resistant malaria in Africa [59]. To prevent the emergence of resistant mutants, various drugs have been studied in combination with artemisinin derivatives, according to the underlying principle to combine artemisinins with drugs that have long plasma elimination half-lives. These treatments seems inappropriate for patients from areas with a high rate of malaria transmission because of the increased risk of drug-resistant mutants resulting from prolonged exposure to subtherapeutic levels of the slowly eliminated drug in the combination [7, 60, 61]. In the same way, combination therapy with drugs that have a rapid elimination time reduces the selection of resistant isolates [62]. The difficulty lies in choosing the ideal combination given the pharmacokinetic properties of the molecules used. One clinical trial combining artesunate with clindamycin for the treatment of uncomplicated *P. falciparum* malaria in Gabonese children was reported in 2005 [7]. In this trial, clindamycin was selected based on promising results from animal models, in vitro studies of *P. falciparum* and the use of sequential treatment with artesunate and clindamycin on Brazilian children [63]. An open-labelled, randomized, controlled clinical trial was performed to evaluate the efficacy and tolerance of oral artesunate-clindamycin therapy (2 and 7 mg/kg) administered twice daily for 3 days compared with a standard quinine–clindamycin regimen administered twice daily for 3 days to treat uncomplicated falciparum malaria in 100 children. The results showed that the artesunate-clindamycin combination was consistent with that of quinine–clindamycin with respect to the cure rates (87 versus 94 % at day 28 of follow up). The decreased fever and parasites clearance were significantly shorter in the artesunate-clindamycin treatment group. Based on the results of this study, clindamycin associated with artemisinin-based combination therapy is a candidate for studies in areas with a high rate of malaria transmission.

Another in vivo study was conducted to evaluate the efficacy and drug interactions of clindamycin in combination with other anti-malarial drugs in populations from endemic areas. Some artemisinin derivatives have been tested on mice, such as the novel semi-synthetic endoperoxide artemisone [64]. This compound is synthetized from dihydroartemisin in a one-step process and in combination with clindamycin, exhibited increased antiplasmodial activity, improved in vivo half-life, improved oral bioavailability and metabolic stability, and presented tolerance and no neurotoxicity in humans compared with artesunate. Because this drug is a good candidate, clinical studies must be performed to assess the effect of artemisone in combination with other anti-malarials. If macrolides and their derivatives have been considered as good candidates for the treatment of uncomplicated malaria, their pharmacokinetic properties make them inconsistent against malaria in monotherapy [39].

### Resistance mechanisms

Resistance to macrolides and lincosamides has been increasingly reported in clinical isolates of Gram-positive bacteria. One aspect of this resistance is the multiplicity of mechanisms and the diversity in phenotypic expression of several of these mechanisms. Bacteria resist macrolides and lincosamides antibiotics in three ways, including target-site modification through methylation or mutation to prevent the binding of the antibiotics to ribosomal targets, which confers broad-spectrum resistance to macrolides and lincosamides, antibiotic efflux, and drug inactivation. However, these last two mechanisms only affect some molecules [65].

Ribosomal methylation remains the most widespread mechanism of resistance. Resistance to erythromycin has been observed in staphylococci since 1956. Biochemical studies indicated that resistance resulted from the methylation of the ribosomal target of the antibiotics, leading to cross-resistance to macrolides, lincosamides and streptogramin B. Subsequently, the MLS_B_ phenotype encoded by a variety of *erm* (erythromycin ribosome methylase) genes was reported in a large number of microorganisms [66]. Erm proteins facilitate the dimethylation of a single adenine in nascent 23S rRNA, as a part of the large (50S) ribosomal subunit [67]. A wide range of microorganisms, including spirochetes and anaerobes, which express Erm methylases, are targets for macrolides and lincosamides. The target mutation was first described for *Escherichia coli* mutants highly resistant to erythromycin. Mutations in domain V of rRNA were identified in 2001 [68]. Depending on the species, bacteria possess 1 to several *rrn* operons encoding 23S rRNA. In general, these mutations were observed in pathogens with 1 or 2 *rrn* copies, often with each copy carrying the mutation. This mechanism is responsible for the clarithromycin resistance of some *Mycobacterium avium*, *Helicobacter pylori* and *Treponema pallidum* strains [65]. Mutations in ribosomal proteins L4 and L22, which confer erythromycin resistance, have been documented for *Streptococcus pneumoniae*.

The antibiotic efflux is the second mechanism of resistance described for macrolides. In Gram-negative bacteria, chromosomally encoded pumps contribute to intrinsic resistance to hydrophobic compounds, such as macrolides [66, 69]. These pumps often belong to a family comprising proteins with 12 membrane-spanning regions [70]. In Gram-positive organisms, the acquisition of macrolide resistance through active efflux is mediated through two classes of pumps: the ATP-binding-cassette (ABC) transporter superfamily and the major facilitator superfamily (MFS) [71]. The genes encoding these pumps are variable depending on the bacterial genus. The efflux system is multicomponent in nature, involving plasmidic and chromosomal genes that constitute a fully operational efflux pump with specificity for 14- and 15-membered macrolides and type B streptogramines (the MS_B_ phenotype).

The last mechanism of bacterial resistance is the inactivation of antibiotics. Esterases and phosphotransferases reported in enterobacteria confer resistance to erythromycin and other 14- and 15-membered macrolides, but not to lincosamides. These resistance mechanisms have not been considered of major clinical importance because enterobacteria are not targets for macrolides. Some clinical isolates of *S. aureus* produce phosphotransferases, but this event remains rare [72–74]. In pathogenic microorganisms, the impact of the three mechanisms is unequal in terms of incidence and clinical implications.

Concerning *Plasmodium* spp., if prophylactic failures have been observed neither for both the two molecules, in vivo resistance has not been demonstrated nor for clindamycin or azithromycin. However, experimental models of resistant *Plasmodium* have been developed under selection pressure: strains of *P. berghei* resistant to clindamycin were described in two studies performed in the 1970s [75, 76]; *P. falciparum* isolates resistant to azithromycin have been developed later [16], but mechanisms underlying the resistance of *Plasmodium* against molecules from the MLSB family were not clearly identified. Mutations on A1875 (corresponding to the *E. coli* A2058 nucleotide in the peptidyltransferase centre of domain V) and A706 (corresponding to the *E. coli* A754 in domain II) in the *P. falciparum* apicoplast LSU rRNA (bearing 70 % identity to the 23S rRNA [77] did not confer in vitro resistance to macrolide in *P. falciparum* as observed in bacterial species [16]. The G1878 mutation, which confers resistance to clindamycin and azithromycin in *Toxoplasma gondii* [33], remained unchanged in azithromycin-resistant *P. falciparum* parasites [16]. A mutation was identified at nucleotide position 438 (T438C) after azithromycin-resistance selection. A single point mutation was also identified at codon 76 (G76 V) in the *Pfrpl4* gene in azithromycin-resistant parasites.

## Conclusions

The emergence and rapid extension of *P. falciparum* resistance to principal anti-malarial drugs necessitates the search for new molecules. Macrolides and their derivatives have been considered as good candidates but the design of more effective structural analogues is required, essentially to improve pharmacokinetic properties. The synthesis of single compounds that yields both fast- and slow-acting profiles by targeting different parasite metabolic processes is being developed to achieve effective molecules and mitigate parasite resistance.
